# Liver transplantation for intrahepatic cholangiocarcinoma: Insights from a National Registry Study in China

**DOI:** 10.1097/MD.0000000000044942

**Published:** 2025-10-17

**Authors:** Pengcheng Wei, Delin Ma, Chen Lo, Yongjing Luo, Jie Gao, Lei Huang, Jiye Zhu, Guangming Li, Shusen Zheng, Zhao Li

**Affiliations:** a Department of Hepatobiliary Surgery, Peking University People’s Hospital, Beijing, China; b Beijing Key Surgical Basic Research Laboratory of Liver Cirrhosis and Liver Cancer, Peking University People’s Hospital, Beijing, China; c Peking University Center of Liver Cancer Diagnosis and Treatment, Peking University People’s Hospital, Beijing, China; d Peking University Institute of Organ Transplantation, Peking University People’s Hospital, Beijing, China; e Department of General Surgery Center, Beijing You An Hospital, Clinical Center for Liver Cancer, Capital Medical University, Beijing, China; f China Liver Transplant Registry, Zhejiang University, Hangzhou, China; g National Center for Healthcare Quality Management of Liver Transplant, Zhejiang University, Hangzhou, China; h Department of Hepatobiliary and Pancreatic Surgery, The First Affiliated Hospital, Zhejiang University School of Medicine, Hangzhou, China; i NHC Key Laboratory of Combined Multi-organ Transplantation, Zhejiang University, Hangzhou, China; j Institute of Organ Transplantation, Zhejiang University, Hangzhou, China.

**Keywords:** intrahepatic cholangiocarcinoma, liver transplantation, neoadjuvant therapy, prognosis, survival analysis

## Abstract

This study investigates the clinical efficacy of liver transplantation (LT) for intrahepatic cholangiocarcinoma (ICC), identifies clinicopathologic factors impacting prognosis, and provides evidence-based data to support LT indications for ICC. A retrospective cohort study was conducted using data from 124 ICC patients in the Chinese Liver Transplant Registry from January 2015 to December 2020. Clinicopathologic data and survival outcomes were analyzed. Postoperative survival was assessed using Kaplan–Meier analysis and Log-rank tests, while Cox regression identified prognostic factors. The number of LT cases for ICC increased yearly from 2015 to 2019, with major centers located in Shanghai, Guangdong, Beijing, Zhejiang, and Hubei. The overall survival (OS) rates at 1, 3, and 5 years were 68.2%, 35.5%, and 32.0%, respectively, and the disease-free survival (DFS) rates were 51.0%, 29.3%, and 26.5%, respectively. Poorer prognosis was linked to male gender, poor Child–Pugh classification, and not meeting the Hangzhou criteria. A significant OS difference was found between 89 male and 35 female patients (*P* = .034), with female patients showing better OS than male patients, but no significant difference was observed in DFS (*P* = .084). Among 78 patients exceeding the Milan criteria, those with preoperative neoadjuvant therapy had better OS (*P* = .041), though DFS differences were not significant (*P* = .228). LT prognosis for ICC is influenced by multiple factors, including worse outcomes for males. Preoperative neoadjuvant therapy improves survival for patients exceeding the Milan criteria but does not significantly prevent recurrence. Optimization of recipient selection and neoadjuvant therapy application is necessary.

## 1. Introduction

Intrahepatic cholangiocarcinoma (ICC), a malignant and aggressive tumor originating from the secondary bile ducts and their branches within the liver, accounts for 10% to 15% of primary liver cancers.^[1–3]^ In recent years, its incidence has significantly increased worldwide, second only to hepatocellular carcinoma.^[4,5]^ The incidence of cholangiocarcinoma in Asia is significantly higher than in European and American populations.^[6]^ Although the etiology of ICC is not fully understood, it is associated with various risk factors, including advanced age, viral hepatitis, choledocholithiasis, primary sclerosing cholangitis, hepatic schistosomiasis, and liver cirrhosis.^[7,8]^ ICC has an insidious onset, progresses rapidly, and is prone to invading peripheral hepatic tissues, organs, nerves, lymph nodes, and causing extrahepatic distant metastasis. Early-stage ICC often has no specific symptoms, leading to most patients being diagnosed at an advanced stage, where effective treatment options are lacking.^[9,10]^

Currently, radical hepatic resection (LR) is still considered the only potential cure for ICC, but only 20% to 30% of patients are eligible for LR, and the 5-year survival rate post-surgery is only 25% to 40%.^[10–12]^ Due to the low resection rate, poor prognosis, and high recurrence rate of ICC patients, liver transplantation (LT) has gained attention as a treatment. Whether LT can achieve better outcomes than LR is a current research focus. Theoretically, LT can completely remove intrahepatic micrometastases and reduce positive cholangiocutaneous margins. However, early studies showed low long-term survival and high recurrence rates after LT in ICC patients, leading to its initial classification as a contraindication for LT.^[13,14]^ However, with accumulating experience and increasing volumes of LT for ICC, more clinical studies have questioned this traditional view.^[15]^ For highly selected ICC patients, LT alone or combined with neoadjuvant therapy has shown better efficacy, making ICC a relative indication for LT.^[16,17]^

In this study, we retrospectively analyzed the clinicopathological data of ICC patients treated with LT in the China Liver Transplant Registry (CLTR) in recent years. Our aims were to explore the clinical efficacy of LT for ICC, identify clinicopathological factors affecting patient prognosis, and provide new evidence-based medical evidence for LT indications in ICC. This will guide clinical treatment decisions and improve overall patient prognosis.

## 2. Materials and methods

### 2.1. Study population

**Figure 1.**
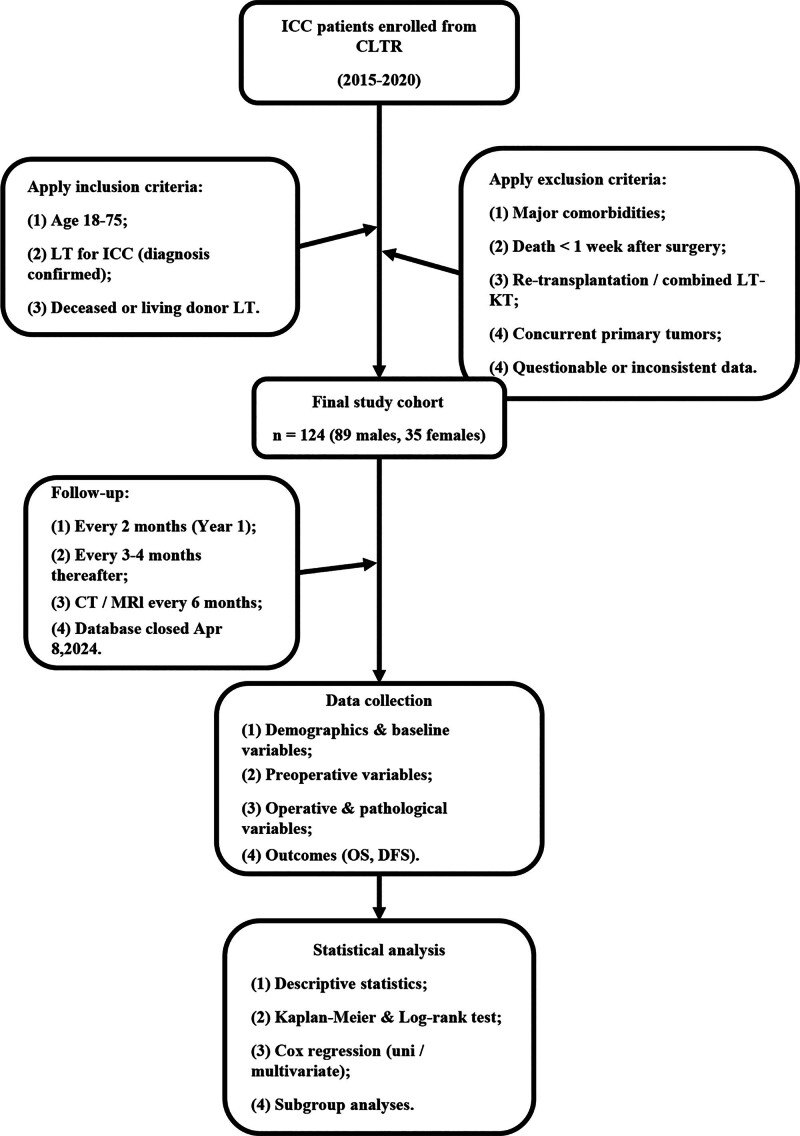
Study flow chart of patients. CLTR = China Liver Transplantation Registry; CT = computed tomography; DFS = disease-free survival, ICC = intrahepatic cholangiocarcinoma, LT = liver transplantation, LT–KT = combined liver–kidney transplantation, MRI = magnetic resonance imaging, OS = overall survival.

This study included 124 ICC patients (89 males and 35 females) enrolled in the CLTR from January 2015 to December 2020, selected using a retrospective cohort study. Inclusion criteria included ICC recipients with a preoperative clinical diagnosis confirmed by postoperative pathology, aged 18 to 75 years, and those who underwent LT from deceased Chinese citizens or living donors. Exclusion criteria included recipients with a history of cardiac, cerebrovascular, or pulmonary diseases, hypertension, or diabetes; death within 1 week post-surgery; re-LT; combined liver and kidney transplantation; concurrent primary tumors; and questionable data such as suspected duplicates, abnormal recipient age, contradictory survival status, or abnormal/missing graft type. This study was approved by the CLTR Scientific Committee and conducted following the ethical guidelines of the 1975 Declaration of Helsinki, revised in 2013. All patients provided written consent for the use of their clinicopathological information in scientific research. This study adhered to the STROBE (Strengthening the Reporting of Observational Studies in Epidemiology) criteria for retrospective cohort studies. The overall study flow is illustrated in Figure 1.

### 2.2. Data collection

The data for this study were independently extracted by CLTR researchers, who were blinded to the study design. This study recorded the types of transplants, the number of transplants per year, and the regional distribution of transplants in ICC patients nationwide. Observations included age, gender, blood group, BMI, history of hepatitis B virus infection, history of hepatitis C virus infection, history of bile duct stones, history of biliary tract infections, preoperative carcinoembryonic antigen (CEA), carbohydrate antigen 19-9 (CA19-9), AFP levels, model for end-stage liver disease (MELD) score, preoperative liver function (Child–Pugh grading), preoperative waiting time, preoperative TNM staging, number of tumors, maximum tumor diameter, compliance with Milan and Hangzhou criteria, cold ischemia time, postoperative pathological grading, presence of lymph node metastasis, intravascular cancer embolism, intrahepatic metastasis, extrahepatic metastasis, preoperative neoadjuvant therapy, postoperative survival, and recurrence. TNM staging was based on the 8th edition of the AJCC/TNM staging system.

### 2.3. Patient follow-up

ICC patients who met the inclusion criteria were regularly monitored by CLTR for postoperative survival and recurrence, with follow-up until the database cutoff time or patient death. In the first year after LT, recipients underwent routine serologic tests for liver function and tumor markers every 2 months, and at least every 3 to 4 months thereafter. Abdominal computed tomography and magnetic resonance imaging were performed every 6 months and immediately when recurrence was suspected. The database for this study was closed on April 8, 2024.

### 2.4. Statistical analysis

SAS software version 9.4 (SAS Institute, Cary) and SPSS version 22.0 (IBM Corp., Armonk) were used for statistical analysis. Count data were analyzed using absolute numbers and percentages. Differences between groups were analyzed with the χ^2^ test or Fisher’s exact probability method. Postoperative survival was analyzed using the Kaplan–Meier method and survival curves were plotted. Differences in survival between groups were compared using the Log-rank test. Factors influencing survival were analyzed using multivariate COX regression. A *P*-value < .05 indicated statistical significance.

## 3. Results

### 3.1. Time trends and geographic distributions

**Figure 2.**
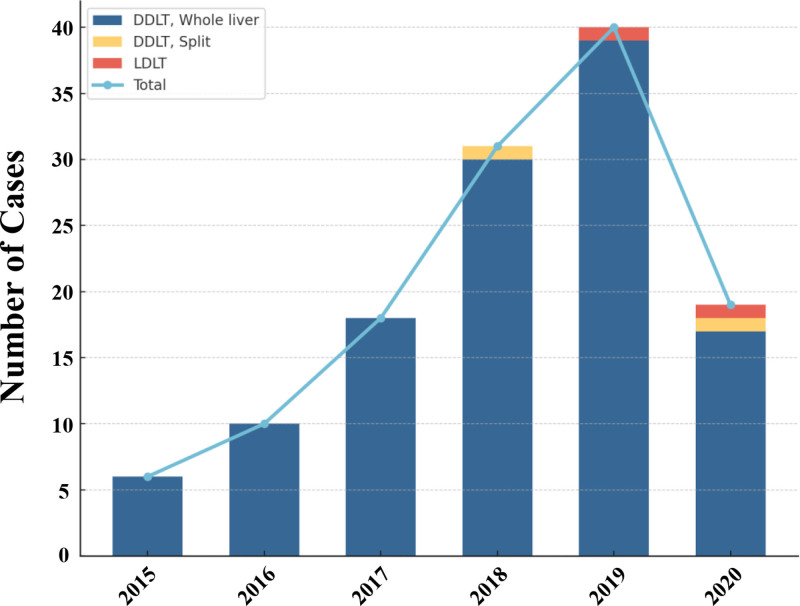
Annual caseloads of liver transplantation for intrahepatic cholangiocarcinoma (ICC) from 2015 to 2020. ICC = intrahepatic cholangiocarcinoma.

A total of 124 ICC patients were enrolled in the CLTR between January 2015 and December 2020. Deceased donor LT was the predominant graft type, whereas living donor LT was rarely performed (Table 1). The annual number of LT cases for ICC steadily increased from 2015 to 2019, peaking in 2019, but subsequently declined in 2020, likely due to the COVID-19 pandemic (Fig. 2). Regarding geographic distribution, the majority of cases were contributed by transplant centers in Shanghai, Guangdong, Beijing, Zhejiang, and Hubei, together accounting for over 70% of the cohort (Table 2).

**Table 1:** Annual caseloads of LT for ICC according to different graft types.

Year	Graft types (number of cases)			
	DDLT, whole liver	DDLT, split	LDLT	Overall
2015	6 (4.8%)	0	0	6 (4.8%)
2016	10 (8.1%)	0	0	10 (8.1%)
2017	18 (14.5%)	0	0	18 (14.5%)
2018	30 (24.2%)	1 (0.8%)	0	31 (25%)
2019	39 (31.5%)	0	1 (0.8%)	40 (32.3%)
2020	17 (13.7%)	1 (0.8%)	1 (0.8%)	19 (15.3%)
Overall	120 (96.8%)	2 (1.6%)	2 (1.6%)	124

DDLT = deceased donor liver transplant, ICC = intrahepatic cholangiocarcinoma, LDLT = living donor liver transplant, LT = liver transplantation.

**Table 2:** The geographic distribution of cases in terms of liver transplantation for intrahepatic cholangiocarcinoma (ICC) from 2015 to 2020.

	2015	2016	2017	2018	2019	2020	Overall
Beijing	0	2 (1.6%)	5 (4%)	4 (3.2%)	3 (2.4%)	1 (0.8%)	15 (12.1%)
Guangdong	1 (0.8%)	1 (0.8%)	3 (2.4%)	6 (4.8%)	10 (8.1%)	5 (4%)	26 (21%)
Guizhou	0	0	0	0	1 (0.8%)	1 (0.8%)	2 (1.6%))
Henan	0	1 (0.8%)	0	0	1 (0.8%)	0	2 (1.6%)
Heilongjiang	0	0	0	0	1 (0.8%)	0	1 (0.8%)
Hubei	2 (1.6%)	1 (0.8%)	0	2 (1.6%)	4 (3.2%)	1 (0.8%)	10 (8.1%)
Hunan	0	0	0	1 (0.8%)	0	0	1 (0.8%)
Jiangsu	0	0	1 (0.8%)	1 (0.8%)	1 (0.8%)	3 (2.4%)	6 (4.8%)
Jiangxi	0	0	2 (1.6%)	1 (0.8%)	1 (0.8%)	0	4 (3.2%)
Shandong	0	0	2 (1.6%)	1 (0.8%)	3 (2.4%)	0	6 (4.8%)
Shanxi	1 (0.8%)	0	0	1 (0.8%)	2 (1.6%)	0	4 (3.2%)
Shanghai	2 (1.6%)	3 (2.4%)	2 (1.6%)	8 (6.5%)	8 (6.5%)	4 (3.2%)	27 (21.8%)
Sichuan	0	1 (0.8%)	1 (0.8%)	0	2 (1.6%)	2 (1.6%)	6 (4.8%)
Tianjin	0	0	0	0	0	1 (0.8%)	1 (0.8%)
Zhejiang	0	1 (0.8%)	2 (1.6%)	5 (4%)	3 (2.4%)	1 (0.8%)	12 (9.7%)
Chongqing	0	0	0	1 (0.8%)	0	0	1 (0.8%)
Overall	6 (4.8%)	10 (8.1%)	18 (14.5%)	31 (25%)	40 (32.3%)	19 (15.3%)	124

ICC = intrahepatic cholangiocarcinoma.

### 3.2. Clinicopathological characteristics of the study cohort

**Figure 3.**
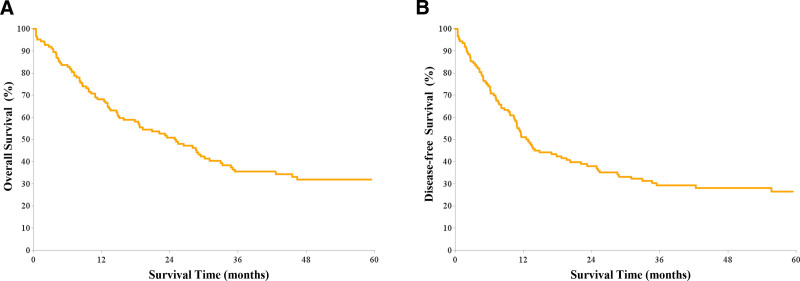
Overall survival curve and disease-free survival curve after surgery in 124 patients with intrahepatic cholangiocarcinoma (ICC) in the study cohort. (A) Overall survival curve; (B) Disease-free survival curve. ICC = intrahepatic cholangiocarcinoma.

Among the 124 ICC patients, the majority were male, and nearly 1-quarter were older than 60 years. Hepatitis B virus infection and cirrhosis were the most common comorbidities, while a minority had hepatitis C virus infection, choledocholithiasis, or biliary tract infections. Half of the patients had a preoperative MELD score > 18, and approximately 40% had advanced TNM stage (≥III). Detailed clinicopathological characteristics are presented in Table 3. Regarding survival outcomes, the overall survival (OS) rates at 1, 3, and 5 years were 68.2%, 35.5%, and 32.0%, respectively, while the disease-free survival (DFS) rates were 51.0%, 29.3%, and 26.5%, respectively. The corresponding Kaplan–Meier survival curves are illustrated in Figure 3.

**Table 3:** Clinicopathological characteristics of the study cohort.

Influencing factors	n (%)
Age (yr) (<60:≥60)	94:30 (75.8:24.2)
Sex (female:male)	35:89 (28.2:71.8)
Blood type (A:B:AB:O)	31:41:15:37 (25:33.1:12.1:29.8)
BMI (kg/m^2^) (<18.5:18.5–24:≥24)	9:77:38 (7.3:62.1:30.6)
History of hepatitis B (none:yes)	75:49 (60.5:39.5)
History of hepatitis C (none:yes)	120:4 (96.8:3.2)
History of bile duct stones (none:yes)	121:3 (97.6:2.4)
History of cirrhosis (none:yes)	45:79 (36.3:63.7)
History of biliary tract infection (none:yes)	123:1 (99.2:0.8)
Preoperative CEA (ng/mL) (<4.7:≥4.7:NA)	44:21:59 (35.5:16.9:47.6)
Preoperative CA19-9 (IU/mL) (<39:39 to 183:≥183:NA)	28:13:23:60 (22.6:10.5:18.5:48.4)
Preoperative AFP (ng/mL) (<25:≥25:NA)	91:23:10 (73.4:18.5:8.1)
Preoperative MELD score (<12:12–18 ≥18)	46:16:62 (37.1:12.9:50)
Child–Pugh classification of liver function (A:B:C)	27:40:57 (21.8:32.3:46)
Preoperative waiting time (mo) (<12:≥12:NA)	84:34:6 (67.7:27.4:4.8)
Preoperative TNM staging (stage I–II:stage III and above:NA)	63:50:11 (50.8:40.3:8.9)
Number of tumors (single:multiple:NA)	65:46:13 (52.4:37.1:10.5)
Maximum tumor diameter (cm) (<2:2 to 5:≥5:NA)	9:52:57:6 (7.3:41.9:46:4.8)
Whether meet Milan criteria (yes:none:NA)	38:78:8 (30.6:62.9:6.5)
Whether meet Hangzhou criteria (yes:none:NA)	62:45:17 (50:36.3:13.7)
Cold ischemia time (h) (<6:≥6:NA)	49:72:3 (39.5:58.1:2.4)
Postoperative pathological grading (degree of differentiation) (high:medium:low:NA)	8:25:10:81 (6.5:20.2:8.1:65.3)
Lymph node metastasis (none:yes)	116:8 (93.5:6.5)
Intravascular cancer embolism (none:yes)	93:31 (75:25)
Intrahepatic metastasis (none:yes:NA)	21:46:57 (16.9:37.1:46)
Extrahepatic metastasis (none:yes)	10:114 (8.1:91.9)
Preoperative neoadjuvant therapy (none:yes)	98:26 (79:21)
Survival (survived:died:lost to visit)	45:72:7 (36.3:58.1:5.6)
Recurrence (recurrence:no recurrence)	50:74 (40.3:59.7)

NA = indicates that data is missing.

AFP = alpha-fetoprotein, BMI = body mass index, CA19-9 = carbohydrate antigen 19-9, CEA = carcinoembryonic antigen, MELD = model for end stage liver disease.

### 3.3. Univariate analysis of OS and DFS in ICC patients post liver transplantation

Univariate analysis showed that several clinicopathological factors were significantly associated with OS and DFS after LT (Table 4). Preoperative MELD score, number of tumors, and compliance with the Hangzhou criteria were significant predictors of 1-year OS, while Child–Pugh classification and Hangzhou criteria significantly influenced 1-year DFS.

**Table 4:** Univariate analysis of 1, 3, 5-year overall survival (OS) and disease-free survival (DFS) after liver transplantation in 124 patients with intrahepatic cholangiocarcinoma (ICC).

Variable	Univariate analysis of 1-yr OS and DFS						Univariate analysis of 3-yr OS and DFS						Univariate analysis of 5-yr OS and DFS					
	OS (%)	χ^*2*^ value	*P* value	DFS (%)	χ^*2*^ value	*P* value	OS (%)	χ^*2*^ value	*P* value	DFS (%)	χ^*2*^ value	*P* value	OS (%)	*χ^2^* value	*P* value	DFS (%)	χ^*2*^ value	*P* value
Sex (male:female)	65.87:74.29	1.2121	.2709	46.19:62.86	2.5858	.1078	28.77:52.02	5.0988	.0239	23.26:44.23	4.0616	.0439	27.25:43.35	4.0561	.0440	23.26:34.83	2.9917	.0837
Age (yr) (≥60:<60)	60.00:70.90	1.2864	.2567	36.67:55.70	3.3207	.0684	25.45:38.37	1.5198	.2177	19.44:32.67	3.1440	.0762	25.45:33.94	1.1769	.2780	19.44:28.90	2.7739	.0958
BMI (kg/m^2^) (≥24:18.5–24:<18.5)	81.58:63.10:55.56	4.7528	.0929	65.59:45.88:33.33	5.3951	.0674	43.46:33.75:13.89	2.9812	.2252	38.53:27.03:11.11	4.6961	.0956	39.84:29.78:13.89	3.0099	.2220	33.03:24.95:11.11	4.4003	.1108
Blood type (A:B:AB:O)	74.19:55.89:80.00:72.22	3.8371	.2796	51.08:43.54:60.00:55.56	1.6596	.6459	32.01:28.08:37.04:45.40	4.2905	.2318	24.08:24.83:23.33:40.94	2.6787	.4439	32.01:–:–:45.40	4.8511	.1830	16.05:–:–:37.79	2.6013	.4573
History of hepatitis B (yes:none)	77.47:64.78	0.9176	.3381	56.88:47.09	1.3687	.2420	43.51:30.31	2.0337	.1538	37.44:24.19	3.1914	.0740	37.71:28.42	1.5762	.2093	37.44:20.09	3.8404	.0500
History of hepatitis C (yes:none)	75.00:68.00	0.1121	.7378	50.00:51.01	0.0014	.9700	75.00:33.94	1.7154	.1903	50.00:28.51	0.4353	.5094	75.00:30.26	2.0180	.1554	50.00:25.45	0.5651	.4522
History of bile duct stones (yes:none)	33.33:69.10	1.9035	.1677	33.33:51.42	1.1828	.2768	0.00:36.36	5.6965	–	0.00:30.06	4.0692	–	0.00:32.80	5.6965	–	0.00:27.16	4.0692	–
History of cirrhosis (yes:none)	67.04:70.38	0.1263	.7223	51.71:49.72	0.0263	.8711	38.25:29.32	0.1699	.6802	33.58:21.79	0.8422	.3588	35.19:24.43	0.2502	.6169	29.70:21.79	0.6958	.4042
History of biliary tract infection (yes:none)	100.00:67.97	0.3846	.5352	0.00:51.42	0.3790	.5381	–:35.19	0.6435	.4225	0.0:29.56	0.3790	.5381	–:31.75	0.6435	.4225	0.00:26.71	0.3790	.5381
Preoperative AFP (ng/mL) (≥25:<25)	73.91:67.72	0.3358	.5623	46.82:52.08	0.0224	.8811	46.20:34.73	0.6375	.4246	37.46:28.61	0.3331	.5639	46.20:29.68	1.0988	.2945	37.46:26.93	0.4276	.5132
Preoperative CA19-9 (IU/mL) (≥183:39–183:<39)	69.57:60.58:64.29	0.3476	.8405	52.17:51.92:42.86	1.2961	.5231	31.79:51.92:32.14	1.1051	.5755	25.36:43.27:25.00	1.8509	.3963	25.43:43.27:28.13	0.9303	.6280	25.36:43.27:20.83	2.2320	.3276
Preoperative CA19-9 (IU/mL) (≥39:<39)	66.44:64.29	0.2556	.6132	52.50:42.86	1.1837	.2766	39.21:32.14	0.5500	.4583	31.82:25.00	0.7935	.3731	32.08:28.13	0.4182	.5178	31.82:20.83	1.1091	.2923
Preoperative CA19-9 (IU/mL) (≥183:<183)	69.57:63.32	0.3086	.5785	52.17:45.90	0.1605	.6887	31.79:37.99	0.0079	.9292	25.36:30.60	0.0724	.7879	25.43:32.56	0.0192	.8898	25.36:27.82	0.0402	.8412
Preoperative CEA (ng/mL) (≥4.7:<4.7)	57.14:68.18	0.2487	.6180	42.86:49.72	0.2894	.5906	21.43:42.19	1.5224	.2173	17.86:32.83	1.3826	.2397	–:36.92	1.8811	.1702	–:30.09	1.2587	.2619
Preoperative MELD score (≥18:12–18:<12)	70.97:93.33:56.27	6.7345	.0345	54.84:53.33:44.98	1.3220	.5163	45.05:7.62:31.99	3.7769	.1513	33.01:6.67:33.26	1.8094	.4047	38.29:7.62:31.99	2.8636	.2389	30.01:6.67:29.94	1.7526	.4163
Child–Pugh classification of liver function (A:B:C)	69.96:58.97:73.68	2.8418	.2415	62.09:35.90:56.14	6.6875	.0358	45.68:26.32:37.36	4.9368	.0847	46.57:20.51:27.57	7.1223	.0284	45.58:26.32:29.89	4.4374	.1087	41.39:20.51:23.64	6.1529	.0461
Preoperative waiting time (months) (≥12:<12)	63.36:70.20	0.2683	.6045	51.04:51.00	0.0637	.8008	32.75:36.52	0.0095	.9224	25.80:31.62	0.0001	.9943	27.30:33.27	0.0387	.8440	19.35:29.95	0.0196	.8886
Preoperative TNM staging (stage I-II:stage III and above)	68.15:63.27	0.1889	.6639	52.03:46.94	0.6194	.4313	38.61:28.54	1.0044	.3162	32.66:23.70	1.3089	.2526	34.75:25.37	0.9777	.3228	27.55:23.70	0.9716	.3243
Maximum tumor diameter (cm) (≥5:2–5:<2)	64.29:74.76:44.44	4.3656	.1127	44.64:57.01:44.44	2.1607	.3395	27.38:37.80:44.44	2.6900	.2605	24.63:27.86:44.44	1.0270	.5984	27.38:33.07: –	2.0610	.3568	24.63:21.89: –	0.7408	.6905
Number of tumors (single:multiple)	76.43:58.70	3.9235	.0476	55.75:43.48	2.1384	.1436	38.06:29.57	1.9440	.1632	33.60:23.04	2.0078	.1565	35.68:26.88	2.0032	.1570	31.20:18.43	2.1449	.1430
Whether meet Milan criteria (yes:none)	76.14:64.91	1.6272	.2021	57.47:46.59	1.4652	.2261	38.56:32.71	1.3850	.2393	31.99:26.01	1.1735	.2787	35.06:28.86	1.3980	.2371	31.99:21.34	1.5507	.2130
Whether meet Hangzhou criteria (yes:none)	80.23:51.01	10.6931	.0011	58.71:39.77	5.3579	.0206	39.42:30.65	3.8962	.0484	32.21:22.81	3.4956	.0615	35.04:27.24	3.6680	.0555	28.63:19.55	3.7337	.0533
Cold ischemia time (hours) (≥6:<6)	68.87:66.00	0.0679	.7945	54.57:44.00	0.7394	.3898	39.77:28.21	1.3097	.2524	33.57:23.57	1.0787	.2990	35.98:25.08	1.2752	.2588	31.60:19.64	1.2093	.2715
Postoperative pathological grading (degree of differentiation) (high:medium:low)	75.00:64.00:70.00	0.4730	.7894	50.00:39.60:40.00	0.9910	.6093	50.00:51.20:46.67	0.1784	.9146	50.00:30.17:40.00	1.3976	.4972	50.00:38.40:46.67	0.5004	.7787	50.00:22.63:40.00	1.8201	.4025
Lymph node metastasis (yes:none)	75.00:67.77	0.1844	.6676	50.00:51.07	0.0046	.9457	15.00:36.81	0.3300	.5657	0.00:31.65	1.4545	.2278	0.00:34.36	0.9781	.3227	0.00:28.59	1.4545	.2278
Intravascular cancer embolism (yes:none)	54.84:72.76	3.5749	.0587	41.69:54.15	1.9675	.1607	36.76:35.19	0.4743	.4910	30.57:28.94	0.3058	.5803	36.76:30.72	0.2312	.6307	30.57:25.31	0.1792	.6720
Intrahepatic metastasis (yes:none)	67.26:80.95	1.2636	.2610	49.60:51.59	0.1589	.6902	41.66:40.98	0.1054	.7455	35.70:29.02	0.0113	.9152	35.71:40.98	0.2788	.5975	32.45:29.02	0.0422	.8373
Extrahepatic metastasis (yes:none)	68.06:70.00	0.0017	.9676	52.85:30.00	2.1570	.1419	36.02:30.00	0.1419	.7064	30.07:20.00	1.3410	.2469	32.11:30.00	0.0588	.8084	26.85:20.00	1.1058	.2930
Preoperative neoadjuvant therapy (yes:none)	69.23:67.93	0.0054	.9417	53.85:50.17	0.1288	.7197	41.77:33.72	0.5362	.4640	34.71:27.74	0.5251	.4687	35.80:30.91	0.4071	.5234	18.51:27.74	0.0875	.7674

AFP = alpha-fetoprotein, BMI = body mass index, CA19-9 = carbohydrate antigen 19-9, CEA = carcinoembryonic antigen, DFS = disease-free survival, ICC = intrahepatic cholangiocarcinoma, MELD = model for end stage liver disease, OS = overall survival.

Notably, female patients had better survival outcomes, with higher 3-year OS (52.0% vs 28.8%, *P* = .024), 3-year DFS (44.2% vs 23.3%, *P* = .044), and 5-year OS (43.4% vs 27.3%, *P* = .044) compared with males. Child–Pugh class A patients demonstrated superior DFS at 1-year (62.1% vs 35.9% and 56.1%, *P* = .036), 3 years (46.6% vs 20.5% and 27.6%, *P* = .028), and 5 years (41.4% vs 20.5% and 23.6%, *P* = .046) compared with those in class B or C. Patients meeting the Hangzhou criteria had higher 1-year OS (80.2% vs 51.0%, *P* = .001), 1-year DFS (58.7% vs 39.8%, *P* = .021), and 3-year OS (39.4% vs 30.6%, *P* = .048) compared with those exceeding the criteria. In addition, patients with a MELD score of 12 to 18 had better 1-year OS (93.3% vs 71.0% and 56.2%, *P* = .035), and those with a single tumor also had superior 1-year OS (76.4% vs 58.7%, *P* = .048). Further details of all univariate analyses are presented in Table 4.

### 3.4. Multivariate analysis of OS and DFS in ICC patients post liver transplantation

Cox multivariate analysis identified several independent prognostic factors for ICC patients after LT (Table 5). Female sex was an independent protective factor, associated with lower risks of death at 3 years (HR reduction 48.9%, *P* = .024) and 5 years (HR reduction 41.7%, *P* = .044), as well as lower recurrence risk at 3 years (HR reduction 46.4%, *P* = .044), compared with males. Meeting the Hangzhou criteria was also an independent factor, with significantly lower risks of death at 1-year (HR reduction 64.8%, *P* = .001), recurrence at 1-year (HR reduction 47.1%, *P* = .021), and death at 3 years (HR reduction 41.2%, *P* = .048), compared with those exceeding the criteria. In addition, Child–Pugh class C liver function was independently associated with increased recurrence risks at 3 years (2.55-fold, *P* = .028) and 5 years (1.96-fold, *P* = .046), compared with Child–Pugh class A or B.

**Table 5:** Multivariate analysis of 1, 3, 5-year overall survival (OS) and disease-free survival (DFS) after liver transplantation in 124 patients with intrahepatic cholangiocarcinoma (ICC).

Variable	1-yr OS		3-yr OS		5-yr OS	
	HR (95% CI)	*P*-value	HR (95% CI)	*P*-value	HR (95% CI)	*P*-value
Sex (female:male)			0.511 (0.277–0.941)	0.031	0.583 (0.344–0.968)	.045
Preoperative MELD score (≥18:<18)	1.390 (0.969–1.995)	.074				
Whether meet Hangzhou criteria (yes:none)	0.352 (0.173–0.714)	.004	0.588 (0.359–0.963)	0.035	0.711 (0.462–1.094)	.120

	1-yr DFS		3-yr DFS		5-yr DFS	
	HR (95% CI)	*P*-value	HR (95% CI)	*P*-value	HR (95% CI)	*P*-value
Sex (female:male)			0.536 (0.318–0.902)	0.019		
History of hepatitis B (none:yes)					0.692 (0.439–1.090)	.113
Child–Pugh classification of liver function (C:A and B)	1.718 (0.803–3.677)	.163	2.551 (1.312–4.958)	0.019	1.964 (1.034–3.734)	.039
Whether meet Hangzhou criteria (yes:none)	0.529 (0.306–0.914)	.023				

DFS = disease-free survival, ICC = intrahepatic cholangiocarcinoma, MELD = model for end stage liver disease, OS = overall survival.

**Figure 4.**
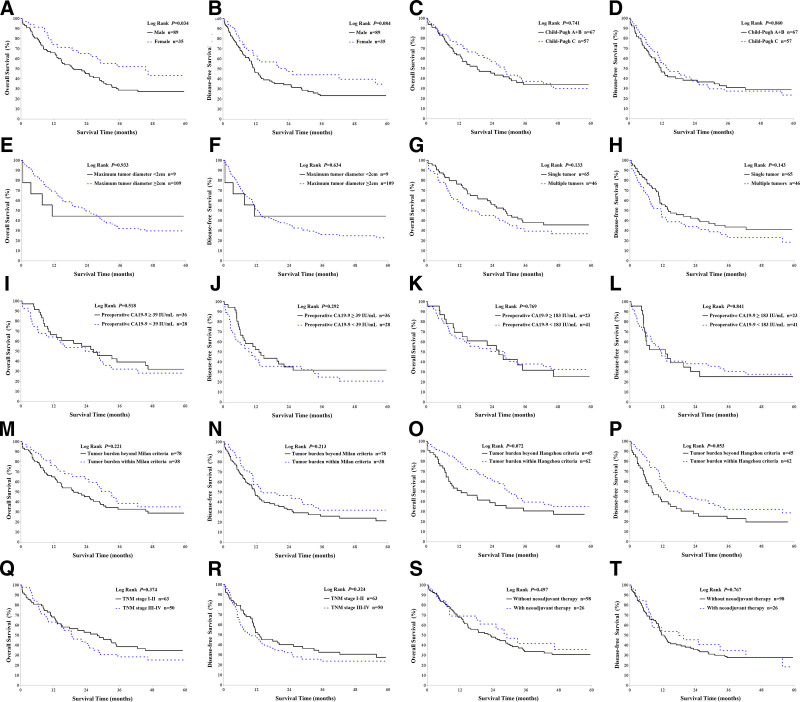
Comparison of overall survival (OS) and disease-free survival (DFS) curves following liver transplantation in intrahepatic cholangiocarcinoma (ICC) patients. (A, B) OS (*P* = .034) and DFS (*P* = .084) for male (n = 89) vs female (n = 35); (C, D) OS (*P* = .741) and DFS (*P* = .860) for Child–Pugh A + B (n = 67) vs Child–Pugh C (n = 57); (E, F) OS (*P* = .933) and DFS (*P* = .634) for tumors < 2 cm (n = 9) vs ≥2 cm (n = 109); (G, H) OS (*P* = .133) and DFS (*P* = .143) for single tumor (n = 65) vs multiple tumors (n = 46); (I, J) OS (*P* = .518) and DFS (*P* = .292) for preoperative CA19-9 ≥ 39 IU/mL (n = 36) vs <39 IU/mL (n = 28); (K, L) OS (*P* = .769) and DFS (*P* = .841) for preoperative CA19-9 ≥ 183 IU/mL (n = 23) vs <183 IU/mL (n = 41); (M, N) OS (*P* = .221) and DFS (*P* = .213) for patients exceeding Milan criteria (n = 78) vs meeting Milan criteria (n = 38); (O, P) OS (*P* = .072) and DFS (*P* = .053) for patients exceeding Hangzhou criteria (n = 45) vs meeting Hangzhou criteria (n = 62); (Q, R) OS (*P* = .374) and DFS (*P* = .324) for early TNM stages (I–II, n = 63) vs advanced TNM stages (III+, n = 50); (S, T) OS (*P* = .497) and DFS (*P* = .767) for patients without neoadjuvant therapy (n = 98) vs with neoadjuvant therapy (n = 26). CA19-9 = carbohydrate antigen 19-9, DFS = disease-free survival, ICC = intrahepatic cholangiocarcinoma, OS = overall survival, TNM = tumor, node, metastasis.

In the survival curve analysis, a significant difference in OS was observed between male and female patients (*P* = .034), while DFS showed no significant difference (*P* = .084). Other subgroup comparisons, including tumor size, number of tumors, CA19-9 levels, Milan criteria, TNM stage, and neoadjuvant therapy, did not demonstrate statistically significant differences and are detailed in Figure 4.

### 3.5. Subgroup analysis of OS and DFS after liver transplantation in ICC patients within and beyond Milan criteria

**Figure 5.**
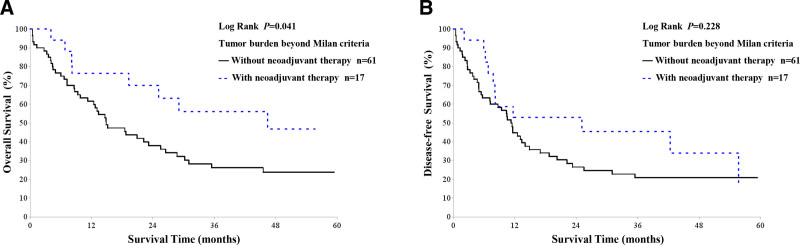
Comparison of overall and disease-free survival curves after liver transplantation in 61 intrahepatic cholangiocarcinoma (ICC) patients without neoadjuvant therapy and 17 patients with neoadjuvant therapy, all exceeding Milan criteria. (A) Overall survival curve; (B) disease-free survival curve. ICC = intrahepatic cholangiocarcinoma.

In the subgroup analysis (Table 6), preoperative neoadjuvant therapy was associated with significantly improved long-term survival. Patients who received neoadjuvant therapy had higher 3-year OS (56.1% vs 26.1%, *P* = .034) and 5-year OS (46.7% vs 23.8%, *P* = .047) compared with those without therapy. Further survival curve analysis showed that among the 78 ICC patients exceeding the Milan criteria, neoadjuvant therapy was associated with significantly better OS (*P* = .041), although no significant difference was observed in DFS (*P* = .228; Fig. 5). These findings suggest that preoperative neoadjuvant therapy may improve survival in ICC patients beyond the Milan criteria, although it does not significantly reduce recurrence risk.

**Table 6:** Subgroup analysis of 1, 3, 5-year overall survival (OS) and disease-free survival (DFS) after liver transplantation in intrahepatic cholangiocarcinoma (ICC) patients within and beyond Milan criteria.

Variable	n	1-yr				3-yr				5-yr			
		OS (%)	*P*-value	DFS (%)	*P*-value	OS (%)	*P*-value	DFS (%)	*P*-value	OS (%)	*P*-value	DFS (%)	*P*-value
Tumor burden within Milan criteria	38												
Preoperative CA19-9			.4969		.8851		.4473		.8061		.5581		.8061
≥39 IU/mL	10	58.33		48.00		23.33		24.00		23.33		24.00	
<39 IU/mL	13	76.92		61.54		46.15		30.77		36.92		30.77	
Preoperative CA19-9			.6077		.8373		.7424		.6991		.8146		.6991
≥183 IU/mL	5	80.00		60.00		20.00		20.00		20.00		20.00	
<183 IU/mL	18	66.20		55.00		42.13		30.56		35.11		30.56	
Preoperative neoadjuvant therapy (yes:none)			.0490		.5076		.0920		.3358		.1110		.3358
Yes	6	50.00		50.00		16.67		16.67		–		–	
None	32	81.02		58.81		42.98		35.00		38.68		35.00	
Tumor burden beyond Milan criteria	78												
Preoperative CA19-9			.2174		.0897		.1259		.1835		.2141		.1196
≥39 IU/mL	23	69.57		52.17		43.48		30.43		32.61		30.43	
<39 IU/mL	15	53.33		26.67		20.00		20.00		20.00		–	
Preoperative CA19-9			.5122		.4563		.6794		.8162		.7593		.7464
≥183 IU/mL	16	68.75		50.00		37.50		25.00		28.13		–	
<183 IU/mL	22	59.09		36.36		31.82		27.27		27.27		22.73	
Preoperative neoadjuvant therapy (yes:none)			.2367		.4012		.0341		.0932		.0471		.2279
Yes	17	76.47		52.94		56.08		45.38		46.73		17.02	
None	61	61.62		44.78		26.13		20.86		23.76		20.86	

CA19-9 = carbohydrate antigen 19-9, DFS = disease-free survival, ICC = intrahepatic cholangiocarcinoma, OS = overall survival.

## 4. Discussion

Early studies have consistently considered ICC a contraindication to LT. Theoretically, LT not only completely removes tumors from the liver but also eliminates underlying microscopic lesions and background liver diseases such as cirrhosis.^[18]^ However, the use of LT as a conventional treatment strategy for ICC has been controversial for a long time. Although many studies on the selection criteria and prognosis of liver transplant recipients for HCC exist, such as the Milan and Hangzhou criteria, there are no standardized recipient selection criteria for ICC LT, and prognostic studies are very limited. Most transplant centers’ studies on ICC LT have small sample sizes, usually fewer than 30 cases. This study included data on 124 ICC patients in the CLTR between 2015 and 2020 and found that the number of ICC patients undergoing LT has increased over time and across regions. This suggests that experience with LT for ICC is gradually accumulating and that more studies will be conducted to challenge traditional concepts.

Identifying factors affecting survival and recurrence after LT in ICC patients is crucial for determining long-term prognosis. A multicenter retrospective study in 2014 showed that ICC patients with a single lesion ≤ 2 cm had a 5-year postoperative OS of more than 60% and a low recurrence rate after LT.^[19]^ A prospective study by McMillan et al demonstrated that ICC patients who underwent LT had a 5-year postoperative OS of 57%. Patients with a single tumor > 2 cm or multiple lesions and a good response to neoadjuvant chemotherapy had a higher long-term postoperative survival rate.^[20]^ A multicenter study by Sapisochin et al reported that the 5-year OS and DFS after LT for very early-stage ICC patients with a single tumor ≤ 2 cm were 65% and 82%, respectively. For patients with progressive ICC with a single tumor > 2 cm or multiple tumors, the 5-year OS and DFS were 45% and 39%, respectively.^[21]^ In this study, the 5-year OS and DFS of ICC patients after LT were 32.0% and 26.5%, respectively. The worse prognosis compared to the above studies may be related to the higher proportion of patients with progressive ICC in this study, where nearly 90% had tumor diameters ≥ 2 cm, about 40% had multiple tumors, more than 40% had preoperative TNM staging of stage III and above, and 63% exceeded the Milan criteria. This may explain the overall lower OS and DFS in this study.

LT can be a relative indication for highly selected ICC patients. Based on previous studies, the International Liver Transplantation Society developed a consensus on LT for ICC treatment in 2020. It recommended that LT may be considered for early-stage ICC patients with a single tumor ≤ 2 cm combined with uncompensated cirrhosis or portal hypertension, or for patients with locally progressive ICC with a sustained response to step down therapy and stable disease.^[22]^ The Spanish Society of Liver Transplantation, in its 2021 consensus, proposed that ICC patients with underlying liver disease not amenable to LR, a single lesion ≤ 2 cm, and no vascular invasion are indications for LT. Conversely, ICC patients with tumor progression (e.g., tumor length > 2 cm, vascular invasion, new lesions, extrahepatic metastases, and significant elevation of CA19-9) while awaiting transplantation, as well as tumor recurrence after LT, are contraindications to LT.^[23]^ This study included a variety of factors that may affect prognosis and recurrence, based on existing domestic and international studies, for unifactorial and multifactorial analysis. The results showed that sex, preoperative MELD score, Child–Pugh classification, number of tumors, meeting Milan criteria, meeting Hangzhou criteria, preoperative neoadjuvant therapy, and other clinicopathological characteristics were associated with long-term survival and tumor recurrence.

In this study, both univariate and multivariate analyses suggest that there are sex differences in the survival prognosis of ICC patients undergoing LT. According to the US National Cancer Institute, the incidence of ICC is higher in men than in women, a trend observed globally.^[24]^ Several clinical studies have indicated that males are an independent risk factor for poorer ICC prognosis, while female ICC patients have lower mortality and recurrence rates than males after surgery.^[25,26]^ This may be related to biological differences, environment and lifestyle factors (e.g., smoking, alcohol consumption, and hepatitis infections are more common in men), and genetic factors. A study by Jackson et al on menopausal hormone therapy and the risk of biliary tract cancers suggests that sex hormones may contribute to the sex differences in ICC prognosis.^[27]^ In this study, 28% of ICC patients were female and 72% were male, about 2.5 times more males than females. Compared to men, female ICC patients had a higher survival rate and lower recurrence rate after LT. This finding is consistent with previous reports in the literature.

In addition, pretransplant neoadjuvant therapy in ICC patients is closely related to prognosis. Neoadjuvant therapies, including chemotherapy, radiotherapy, and local therapy, have been relatively limited in ICC prognosis with insufficient evidence, despite being well studied in HCC. Cercek et al prospectively explored hepatic arterial perfusion of fluorouracil combined with systemic chemotherapy using GEMOX (gemcitabine and oxaliplatin in the treatment of 38 patients with unresectable ICC. Patients with unresectable ICC showed an objective remission rate of 58%, and 4 patients achieved tumor downstaging.^[28]^ Neoadjuvant therapy in ICC not only controls disease progression but also converts some unresectable patients to viable transplant candidates. A study by Lunsford et al with over 7 years of follow-up demonstrated that patients with locally advanced ICC who responded well to preoperative neoadjuvant therapy benefited from LT.^[29]^ A clinical prediction model by Hong et al suggests that neoadjuvant-treated patients with locally advanced ICC have an improved long-term prognosis after LT.^[30]^ Only 26 ICC patients in this study underwent preoperative neoadjuvant therapy, mainly with gemcitabine combined with cisplatin chemotherapy. However, preoperative neoadjuvant therapy had no significant effect on long-term survival and recurrence after LT. This may be related to the low percentage of ICC patients who received neoadjuvant therapy in this study.

However, standardized criteria for LT in ICC are still lacking. In this study, we analyzed the survival curves of all possible correlates of OS and DFS after LT in ICC patients. The results were not statistically significant except for sex. Previous studies have shown that ICC patients who meet the Milan criteria for LT have a higher survival rate than those who exceed the Milan criteria.^[31]^ The Milan criteria are a recognized clinical standard for LT, requiring that the tumor has a single lesion ≤ 5 cm in diameter or up to 3 lesions with a maximum diameter of ≤3 cm, with no intrahepatic macrovascular invasion or distant metastasis.^[32]^ However, in this study, meeting the Milan criteria did not have a statistically significant effect on the prognosis of ICC patients. The Hangzhou criteria were also included in this study and were found to be associated with ICC prognosis in both univariate and multivariate analyses. Proposed by Academician Zheng Shusen’s team in 2008, the Hangzhou criteria first introduced tumor biology and pathology characteristics as selection criteria for liver transplant recipients, marking a breakthrough from previous criteria limited to tumor morphology.^[33]^ However, the Hangzhou criteria are based on AFP, mainly applied to HCC and having little clinical significance for ICC. Drawing on its standard-setting format, we conducted further subgroup analysis of the effects of CA19-9 and neoadjuvant therapy on OS and DFS in ICC patients meeting and exceeding the Milan criteria. The analysis showed that CA19-9 had no statistically significant effect on the prognosis of LT in ICC patients meeting or exceeding the Milan criteria. However, for ICC patients exceeding the Milan criteria, preoperative neoadjuvant therapy could effectively improve survival prognosis but had no significant effect on recurrence prevention.

Over the past decades, significant progress has been made in treating ICC patients with LT. Initially considered contraindicated, LT is now indicated for very early, highly selected patients, and is achieving satisfactory results in patients with locally progressive ICC who respond well to neoadjuvant therapy.^[34]^ Currently, ICC is also evolving in molecular targeted diagnosis and treatment. Based on serology, immunohistochemistry, and imaging, studies targeting genetic abnormalities such as IDH1/2, FGFR2, and BRAF have improved ICC diagnosis accuracy and achieved precise targeted treatment,^[35,36]^ supporting the improvement of LT efficacy. Additionally, tumor proteins such as KRAS, CDKN2A, and TP53 often indicate poor prognosis.^[37]^ Establishing a comprehensive molecular profile can provide more objective recurrence risk stratification and better assessment of transplantation prognosis. Currently, LT for ICC still faces challenges, such as ICC being easily confused with hilar cholangiocarcinoma in differential diagnosis and disease coding.^[38]^ Advancements in clinical measures, radiomics, genomics, and multidisciplinary collaboration can help clarify the diagnosis, optimize liver transplant recipient selection criteria, and develop better treatment strategies.^[39]^ Early circulating tumor DNA sequencing identifies targeted mutations and assesses tumor aggressiveness, while liquid biopsy monitors early recurrence,^[40,41]^ refining risk stratification, guiding targeted neoadjuvant therapy, and improving long-term prognosis. Theoretical and practical experience in ICC LT therapy is accumulating. The development of individualized selection criteria based on molecular typing and imaging histological features is the future direction.^[42]^

In summary, this study investigated the clinical efficacy of LT for ICC, finding that LT combined with neoadjuvant therapy can improve the long-term survival prognosis of some ICC patients. It also summarized various factors affecting OS and DFS after LT for ICC. However, this study has shortcomings, including a retrospective design, small sample size, and short follow-up time. More large-sample, multicenter randomized controlled studies are needed to validate the conclusions and to further summarize clinicopathological information related to the prognosis of LT for ICC. Although the widespread use of LT in the treatment of ICC is still immature, more experience has been accumulated, and significant breakthroughs have been achieved. With the advancement of more prospective trials, more evidence-based medical data will elucidate the significance of LT in treating ICC. This will inform the development of relevant guidelines, improve the long-term prognosis of patients, and reduce the risk of recurrence.

## 5. Conclusions

The prognosis of LT in ICC patients is influenced by several factors, with male patients having a worse prognosis. In ICC patients exceeding the Milan criteria, preoperative neoadjuvant therapy improves survival prognosis but has a limited role in preventing recurrence. Despite advances in LT for ICC, recipient selection criteria and the use of neoadjuvant therapy still need optimization.

## Acknowledgments

We extend our heartfelt gratitude to the staff of the China Liver Transplantation Registry (CLTR) for their invaluable assistance in data acquisition and extraction. We also thank the transplant centers across mainland China for their significant contributions to the CLTR database.

## Author contributions

**Conceptualization:** Pengcheng Wei, Delin Ma, Chen Lo, Yongjing Luo, Jie Gao, Lei Huang, Jiye Zhu, Guangming Li, Shusen Zheng, Zhao Li.

**Data curation:** Pengcheng Wei, Chen Lo, Yongjing Luo, Jie Gao, Lei Huang, Jiye Zhu, Guangming Li, Shusen Zheng, Zhao Li.

**Funding acquisition:** Zhao Li.

**Formal analysis:** Delin Ma.

**Methodology:** Delin Ma.

**Software:** Delin Ma.

**Supervision:** Jie Gao, Lei Huang, Jiye Zhu, Guangming Li, Shusen Zheng, Zhao Li.

**Writing – original draft:** Pengcheng Wei, Delin Ma, Chen Lo, Yongjing Luo, Jie Gao, Lei Huang, Jiye Zhu, Guangming Li, Shusen Zheng, Zhao Li.

**Writing – review & editing:** Pengcheng Wei, Delin Ma, Guangming Li, Shusen Zheng, Zhao Li.

## References

[1] Brindley PJ Bachini M Ilyas SI. Cholangiocarcinoma. Nat Rev Dis Primers. 2021;7:65. 34504109 10.1038/s41572-021-00300-2 PMC9246479

[2] Kelley RK Bridgewater J Gores GJ Zhu AX. Systemic therapies for intrahepatic cholangiocarcinoma. J Hepatol. 2020;72:353–63. 31954497 10.1016/j.jhep.2019.10.009

[3] Wasilewicz MP Becht R. Intrahepatic cholangiocarcinoma-where are we now and where are we going to? Medicina (Kaunas). 2023;59:729. 37109687 10.3390/medicina59040729 PMC10143006

[4] Bragazzi MC Venere R Ribichini E Covotta F Cardinale V Alvaro D. Intrahepatic cholangiocarcinoma: evolving strategies in management and treatment. Dig Liver Dis. 2024;56:383–93. 37722960 10.1016/j.dld.2023.08.052

[5] Valle JW Kelley RK Nervi B Oh D-Y Zhu AX. Biliary tract cancer. Lancet. 2021;397:428–44. 33516341 10.1016/S0140-6736(21)00153-7

[6] Florio AA Ferlay J Znaor A. Global trends in intrahepatic and extrahepatic cholangiocarcinoma incidence from 1993 to 2012. Cancer. 2020;126:2666–78. 32129902 10.1002/cncr.32803 PMC7323858

[7] Barner-Rasmussen N Pukkala E Hadkhale K Färkkilä M. Risk factors, epidemiology and prognosis of cholangiocarcinoma in Finland. United European Gastroenterol J. 2021;9:1128–35. 10.1002/ueg2.12154 PMC8672081 34533900

[8] Clements O Eliahoo J Kim JU Taylor-Robinson SD Khan SA. Risk factors for intrahepatic and extrahepatic cholangiocarcinoma: a systematic review and meta-analysis. J Hepatol. 2020;72:95–103. 31536748 10.1016/j.jhep.2019.09.007

[9] Eletta OA Panayotova GG Lunsford KE. Liver transplant for intrahepatic cholangiocarcinoma. Surg Clin North Am. 2024;104:215–25. 37953037 10.1016/j.suc.2023.07.006

[10] Mazzaferro V Gorgen A Roayaie S Droz Dit Busset M Sapisochin G. Liver resection and transplantation for intrahepatic cholangiocarcinoma. J Hepatol. 2020;72:364–77. 31954498 10.1016/j.jhep.2019.11.020

[11] Moris D Palta M Kim C Allen PJ Morse MA Lidsky ME. Advances in the treatment of intrahepatic cholangiocarcinoma: an overview of the current and future therapeutic landscape for clinicians. CA Cancer J Clin. 2023;73:198–222. 36260350 10.3322/caac.21759

[12] Yui S Yoshikuni K Akinori M. Gradual expansion of the indications for minimally invasive liver resection to include highly complex procedures may improve postoperative outcomes. Mini-invasive Surgery. 2024;8:30.

[13] Emek E Serin A Sahin T. Experience in liver transplantation due to primary sclerosing cholangitis: a single center experience. Transplant Proc. 2019;51:2439–41. 31405746 10.1016/j.transproceed.2019.01.156

[14] Shimoda M Farmer DG Colquhoun SD. Liver transplantation for cholangiocellular carcinoma: analysis of a single-center experience and review of the literature. Liver Transpl. 2001;7:1023–33. 11753904 10.1053/jlts.2001.29419

[15] Panayotova GG Guarrera JV Lunsford KE. Should we reevaluate liver transplantation as an alternative to resection for the treatment of intrahepatic cholangiocarcinoma? Liver Transpl. 2020;26:748–50. 32297472 10.1002/lt.25780

[16] Chen X Du J Huang J Zeng Y Yuan K. Neoadjuvant and adjuvant therapy in intrahepatic cholangiocarcinoma. J Clin Transl Hepatol. 2022;10:553–63. 35836758 10.14218/JCTH.2021.00250 PMC9240234

[17] Sapisochin G Ivanics T Heimbach J. Liver transplantation for intrahepatic cholangiocarcinoma: ready for prime time? Hepatology. 2022;75:455–72. 34859465 10.1002/hep.32258

[18] Vibert E Schwartz M Olthoff KM. Advances in resection and transplantation for hepatocellular carcinoma. J Hepatol. 2020;72:262–76. 31954491 10.1016/j.jhep.2019.11.017

[19] Sapisochin G de Lope CR Gastaca M. Intrahepatic cholangiocarcinoma or mixed hepatocellular-cholangiocarcinoma in patients undergoing liver transplantation: a Spanish matched cohort multicenter study. Ann Surg. 2014;259:944–52. 24441817 10.1097/SLA.0000000000000494

[20] McMillan RR Javle M Kodali S. Survival following liver transplantation for locally advanced, unresectable intrahepatic cholangiocarcinoma. Am J Transplant. 2022;22:823–32. 34856069 10.1111/ajt.16906

[21] Sapisochin G Facciuto M Rubbia-Brandt L. Liver transplantation for “very early” intrahepatic cholangiocarcinoma: international retrospective study supporting a prospective assessment. Hepatology. 2016;64:1178–88. 27481548 10.1002/hep.28744

[22] Sapisochin G Javle M Lerut J. Liver transplantation for cholangiocarcinoma and mixed hepatocellular cholangiocarcinoma: working group report from the ILTS transplant oncology consensus conference. Transplantation. 2020;104:1125–30. 32217937 10.1097/TP.0000000000003212

[23] Rodríguez-Perálvarez M Gómez-Bravo MA Sánchez-Antolín G. Expanding indications of liver transplantation in Spain: consensus statement and recommendations by the Spanish Society of Liver Transplantation. Transplantation. 2021;105:602–7. 32345868 10.1097/TP.0000000000003281

[24] Antwi SO Mousa OY Patel T. Racial, ethnic, and age disparities in incidence and survival of intrahepatic cholangiocarcinoma in the United States; 1995-2014. Ann Hepatol. 2018;17:604–14. 29893702 10.5604/01.3001.0012.0929

[25] Yu T-H Chen X Zhang X-H Zhang E-C Sun C-X. Clinicopathological characteristics and prognostic factors for intrahepatic cholangiocarcinoma: a population-based study. Sci Rep. 2021;11:3990. 33597569 10.1038/s41598-021-83149-5 PMC7889915

[26] Zou Y Xu X Wu T. Sex disparity in clinical characteristics and long-term prognosis after liver resection for patients with intrahepatic cholangiocarcinoma: a propensity score matching analysis. Heliyon. 2024;10:e29910. 38707344 10.1016/j.heliyon.2024.e29910 PMC11066329

[27] Jackson SS Pfeiffer RM Gabbi C Anderson L Gadalla SM Koshiol J. Menopausal hormone therapy and risk of biliary tract cancers. Hepatology. 2022;75:309–21. 34766362 10.1002/hep.32198 PMC8766909

[28] Cercek A Boerner T Tan BR. Assessment of hepatic arterial infusion of floxuridine in combination with systemic gemcitabine and oxaliplatin in patients with unresectable intrahepatic cholangiocarcinoma: a phase 2 clinical trial. JAMA Oncol. 2020;6:60–7. 31670750 10.1001/jamaoncol.2019.3718 PMC6824231

[29] Lunsford KE Javle M Heyne K. Liver transplantation for locally advanced intrahepatic cholangiocarcinoma treated with neoadjuvant therapy: a prospective case-series. Lancet Gastroenterol Hepatol. 2018;3:337–48. 29548617 10.1016/S2468-1253(18)30045-1

[30] Hong JC Petrowsky H Kaldas FM. Predictive index for tumor recurrence after liver transplantation for locally advanced intrahepatic and hilar cholangiocarcinoma. J Am Coll Surg. 2011;212:514–20; discussion 520–1. 21463781 10.1016/j.jamcollsurg.2010.12.005

[31] Huang G Song W Zhang Y Yu J Lv Y Liu K. Liver transplantation for intrahepatic cholangiocarcinoma: a propensity score-matched analysis. Sci Rep. 2023;13:10630. 37391482 10.1038/s41598-023-37896-2 PMC10313647

[32] Mazzaferro V Bhoori S Sposito C. Milan criteria in liver transplantation for hepatocellular carcinoma: an evidence-based analysis of 15 years of experience. Liver Transpl. 2011;17(Suppl 2):S44–57. 21695773 10.1002/lt.22365

[33] Xu X Lu D Ling Q. Liver transplantation for hepatocellular carcinoma beyond the Milan criteria. Gut. 2016;65:1035–41. 25804634 10.1136/gutjnl-2014-308513 PMC4893115

[34] Sun D Lv G Dong J. Liver Transplantation for Intrahepatic Cholangiocarcinoma: What Are New Insights and What Should We Follow? Front Oncol. 2021;11:841694. 35127541 10.3389/fonc.2021.841694 PMC8813740

[35] Komuta M. Intrahepatic cholangiocarcinoma: tumour heterogeneity and its clinical relevance. Clin Mol Hepatol. 2022;28:396–407. 35032970 10.3350/cmh.2021.0287 PMC9293614

[36] Madoff DC Abi-Jaoudeh N Braxton D. An expert, multidisciplinary perspective on best practices in biomarker testing in intrahepatic cholangiocarcinoma. Oncologist. 2022;27:884–91. 35925597 10.1093/oncolo/oyac139 PMC9526481

[37] Boerner T Drill E Pak LM. Genetic determinants of outcome in intrahepatic cholangiocarcinoma. Hepatology. 2021;74:1429–44. 33765338 10.1002/hep.31829 PMC8713028

[38] Selvadurai S Mann K Mithra S Bridgewater J Malik H Khan SA. Cholangiocarcinoma miscoding in hepatobiliary centres. Eur J Surg Oncol. 2021;47(3 Pt B):635–9. 33032867 10.1016/j.ejso.2020.09.039

[39] Chen E-Q Wang M-L Zhang D-M. Plasma apolipoprotein A-V predicts long-term survival in chronic hepatitis B patients with acute-on-chronic liver failure. Sci Rep. 2017;7:45576. 28358016 10.1038/srep45576 PMC5372093

[40] Lapitz A Azkargorta M Milkiewicz P. Liquid biopsy-based protein biomarkers for risk prediction, early diagnosis, and prognostication of cholangiocarcinoma. J Hepatol. 2023;79:93–108. 36868481 10.1016/j.jhep.2023.02.027 PMC10292605

[41] Yarlagadda B Kamatham V Ritter A Shahjehan F Kasi PM. Trastuzumab and pertuzumab in circulating tumor DNA ERBB2-amplified HER2-positive refractory cholangiocarcinoma. npj Precis Oncol. 2019;3:19. 31453370 10.1038/s41698-019-0091-4 PMC6700112

[42] Zheng S-S Yang Z Wu Y-C. Liver transplantation for intrahepatic and perihilar cholangiocarcinoma: current and future. Hepatobiliary Pancreat Dis Int. 2020;19:101–2. 32165068 10.1016/j.hbpd.2020.02.008

